# ShMOLLI and 5(4R) 3 MOLLI T1 mapping in HCM detect similar ECV despite a difference in absolute T1 measurements

**DOI:** 10.1186/1532-429X-17-S1-P326

**Published:** 2015-02-03

**Authors:** Jorge A Gonzalez, Peter Shaw, Yang Yang, Christopher M Kramer, Michael Salerno

**Affiliations:** 1 Cardiology, University of Virginia, Charlottesville, VA, USA; 2 Biomedical Engineering, University of Virginia, Charlottesville, VA, USA; 3 Radiology, University of Virginia, Charlottesville, VA, USA

## Background

T1 mapping and extracellular volume (ECV) measurements enable assessment of diffuse fibrosis in HCM and have been validated against histology. There still remains wide variation in pulse sequences used to measure T1 each with their associated limitations and biases in T1 determination. We sought to compare T1 values and ECV measurements between a 5(4R)3 variant of MOLLI and ShMOLLI in patients undergoing CMR for evaluation of hypertrophic cardiomyopathy (HCM).

## Methods

CMR imaging was performed in 11 patients with HCM on a 1.5T Avanto (Siemens) scanner under an IRB approved protocol. T1 maps were acquired pre contrast, 5, 15 and 30 minutes post 0.15mmol/kg of Gd contrast using both ShMOLLI and MOLLI pulse sequences. Sequence parameters included: TE/TR/FA 1.1 ms/2.5ms/35°, FOV= 340 x 260, resolution 1.8mm x 1.8mm, thickness 8mm. Regions of interest were drawn in the LV blood pool and myocardium and the mean, SD and coefficient of variation T1 values were determined. Partition coefficient (λ) and ECV were determined from all available time points.

Additionally images were acquired in an agarose gel phantom with T1s ranging from 300 to 1400ms. Reference T1s were determined using a spin echo pulse sequence.

## Results

**Figure 1:**
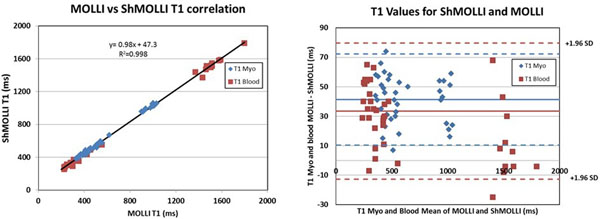


Table 1 shows the comparison of T1s, λ, and ECV between ShMOLLI and MOLLI. T1s differed between techniques for all but the pre-contrast blood measurements. Correlation and Bland Altman Analysis (Fig 1) demonstrate a slope of 0.98 (R^2^=0.99) and a bias of 47 ms (MOLLI>ShMOLLI) across all time points. This bias was similar to the 33 ms bias between the techniques in the phantom experiment. The coefficient of variation of T1 measurements for MOLLI and ShMOLLI were similar (0.063 and 0.031 versus 0.067 and 0.027) for myocardium and blood, respectively, indicating similar uncertainty in the measured T1 times. Notably the λ and ECV were similar between the techniques. With the given bias in T1 measurements a 2% bias in ECV measurements was calculated, explaining why both techniques yield similar ECV despite significant differences in T1.

**Table 1:** Comparison of T1, λ and ECV

	ShMOLLI (msec)	MOLLI (msec)	P-value
T1 Myo (Pre)	964.1 ms (66.9)	1004.6 (72.4)	<0.001

T1 Blood (Pre)	1502.3 (40.4)	1529.6 (39.1)	0.43

T1 Myo (5 min)	385.1 (30)	429.5 (20)	<0.001

T1 Blood (5 min)	262.3 (6.3)	298.5 (10)	<0.001

T1 Myo (15 min)	459 (29.2)	496 (27.6)	<0.001

T1 Blood (15 min)	341.4 (9.6)	355.3 (12.3)	0.03

T1 Myo (30 min)	518.3 (27.9)	422.7 (12.4)	<0.001

T1 Blood (30 min)	556.3 (32.3)	444.1 (13.3)	<0.001

λ (Lambda)	0.49 (0.06)	0.50 (0.06)	0.08

ECV	0.29 (0.03)	0.30 (0.03)	0.08

## Conclusions

T1s from ShMOLLI and MOLLI were highly correlated (R=0.999) however there was a 47ms bias (MOLLI>ShMOLLI) between techniques. Importantly, both methods yielded equivalent ECV measurements in HCM.

## Funding

K23 HL112910 (MS).

T32 EB003841 (JG, PS).

